# Predictive Models for Sustained, Uncontrolled Hypertension and Hypertensive Crisis Based on Electronic Health Record Data: Algorithm Development and Validation

**DOI:** 10.2196/58732

**Published:** 2024-10-28

**Authors:** Hieu Minh Nguyen, William Anderson, Shih-Hsiung Chou, Andrew McWilliams, Jing Zhao, Nicholas Pajewski, Yhenneko Taylor

**Affiliations:** 1 Center for Health System Sciences (CHASSIS), Atrium Health, Charlotte, NC, United States; 2 Statistics and Data Management, Elanco, Greenfield, IN, United States; 3 Enterprise Data Management, Atrium Health, Charlotte, NC, United States; 4 Information Technology, Atrium Health, Charlotte, NC, United States; 5 Department of Internal Medicine, Wake Forest University School of Medicine, Winston-Salem, NC, United States; 6 GSCO Market Access Analytics and Real World Evidence, Johnson & Johnson, Raritan, NJ, United States; 7 Department of Biostatistics and Data Science, Wake Forest University School of Medicine, Winston-Salem, NC, United States; 8 Department of Social Sciences and Health Policy, Wake Forest University School of Medicine, Winston-Salem, NC, United States

**Keywords:** machine learning, risk prediction, predictive model, decision support, blood pressure, cardiovascular, electronic health record

## Abstract

**Background:**

Assessing disease progression among patients with uncontrolled hypertension is important for identifying opportunities for intervention.

**Objective:**

We aim to develop and validate 2 models, one to predict sustained, uncontrolled hypertension (≥2 blood pressure [BP] readings ≥140/90 mm Hg or ≥1 BP reading ≥180/120 mm Hg) and one to predict hypertensive crisis (≥1 BP reading ≥180/120 mm Hg) within 1 year of an index visit (outpatient or ambulatory encounter in which an uncontrolled BP reading was recorded).

**Methods:**

Data from 142,897 patients with uncontrolled hypertension within Atrium Health Greater Charlotte in 2018 were used. Electronic health record–based predictors were based on the 1-year period before a patient’s index visit. The dataset was randomly split (80:20) into a training set and a validation set. In total, 4 machine learning frameworks were considered: L2-regularized logistic regression, multilayer perceptron, gradient boosting machines, and random forest. Model selection was performed with 10-fold cross-validation. The final models were assessed on discrimination (C-statistic), calibration (eg, integrated calibration index), and net benefit (with decision curve analysis). Additionally, internal-external cross-validation was performed at the county level to assess performance with new populations and summarized using random-effect meta-analyses.

**Results:**

In internal validation, the C-statistic and integrated calibration index were 0.72 (95% CI 0.71‐0.72) and 0.015 (95% CI 0.012‐0.020) for the sustained, uncontrolled hypertension model, and 0.81 (95% CI 0.79‐0.82) and 0.009 (95% CI 0.007‐0.011) for the hypertensive crisis model. The models had higher net benefit than the default policies (ie, treat-all and treat-none) across different decision thresholds. In internal-external cross-validation, the pooled performance was consistent with internal validation results; in particular, the pooled C-statistics were 0.70 (95% CI 0.69‐0.71) and 0.79 (95% CI 0.78‐0.81) for the sustained, uncontrolled hypertension model and hypertensive crisis model, respectively.

**Conclusions:**

An electronic health record–based model predicted hypertensive crisis reasonably well in internal and internal-external validations. The model can potentially be used to support population health surveillance and hypertension management. Further studies are needed to improve the ability to predict sustained, uncontrolled hypertension.

## Introduction

Hypertension is a major chronic disease affecting nearly half of the adults in the United States, of whom less than half have their blood pressure (BP) under control [1]. Uncontrolled hypertension, defined as BP ≥140/90 mm Hg may lead to major cardiovascular diseases, organ damage, stroke, or even death, if not properly managed over time [2,3]. Effective treatment for uncontrolled hypertension requires proper monitoring so that further disease progression can be detected and prevented.

Numerous studies have examined the risk factors related to hypertension. Surveillance data show that hypertension is more prevalent in men than in women and in older adults than in younger persons [4]. Racial or ethnic disparities in BP control have been described, owing to risk factors that include racism-related stress, and social barriers such as low health literacy, poverty, and limited access to care [5-7]. Prior studies have also revealed other clinical predictors of hypertension such as comorbidities (eg, coronary heart disease and diabetes), laboratory biomarkers (eg, cholesterol levels), and BMI [8-10]. Leveraging the extensive knowledge base about hypertension risk factors, various prediction models, based on statistical and machine learning methods, have been developed to assess the risk of hypertension onset in the general population [11,12]. However, literature searches revealed a lack of research involving risk prediction for clinically important hypertension states, such as future BP measurements that are consistently elevated, also called sustained uncontrolled hypertension, or hypertensive crisis (ie, BP ≥180/120 mm Hg) in patients with uncontrolled hypertension [13,14].

Predictive models of sustained, uncontrolled hypertension and hypertensive crisis within 1-year following an index visit could inform clinical decision support prompting discussions between patients and clinicians about medication intensification. This index visit can be designated as an outpatient or ambulatory clinic appointment in which the patient had an uncontrolled BP reading and did not have a new antihypertensive medication class added. These specifications can identify a targeted population who may benefit from additional consideration to intensity hypertension medications. From a design perspective, the intended use case for the proposed risk models is to either serve as a real-time nudge that informs a shared decision-making conversation between provider and patient at the index visit or to be deployed as a tool for population health surveillance to help guide timely, proactive outreach. For instance, a care manager may reach out to a patient who is at high risk and did not have medication intensification to schedule a follow-up visit sooner, address barriers to medication adherence, or inquire with the care team about enrolling the patient in a hypertension management program. Furthermore, a valid risk score may aid patients in understanding the importance of treatment decisions; thereby helping to address nonadherence to medications, which is a major risk factor for suboptimal BP control [15]. This study presents the development and validation of 2 risk models, one to predict sustained, uncontrolled hypertension, and one to predict hypertensive crisis within 1 year following an index visit.

## Methods

### Study Population

This study’s cohort consisted of patients aged 18 years or older who had an uncontrolled BP reading (systolic BP ≥140 mm Hg or diastolic BP ≥90 mm Hg) during an ambulatory or outpatient encounter in 2018 at a Greater Charlotte facility of Atrium Health, a large hospital network in the southeastern United States. This study’s cohort only included active patients in the health system, that is, those having at least one encounter during the following year, 2019. The first ambulatory or outpatient encounter during 2018 showing an uncontrolled BP reading was considered the patient’s index visit. Patients were excluded if, on the index visit, they were prescribed a new antihypertensive drug class that was not present in the 1-year historical medication records. Patients on more than 4 drug classes of antihypertensive medication were excluded. Patients were also excluded if they were in hospice care, were pregnant during 2018 or 2019, were diagnosed with end-stage renal disease, received dialysis, had a renal transplant, or died before December 31, 2019.

### Study Variables

Sustained, uncontrolled hypertension in a patient was a binary outcome indicating the presence of ≥2 uncontrolled BP readings (≥140/90 mm Hg) or ≥1 particularly high BP reading (≥180/120 mm Hg) within 1 year following an index visit. The hypertensive crisis outcome was a binary indicator showing whether a patient had any BP reading ≥180/120 mm Hg within 1 year following the index visit.

We determined that using a 1-year look-back window prior to index visit would be appropriate to capture recent, clinically relevant predictor data from the health system’s electronic health records (EHRs). This decision also relied on previous observations of models predicting 1-year hypertension status demonstrating successes when they were also using 1-year-old data prior to prediction [16,17]. We collected basic data at the index visit including the patient’s age, gender, race and ethnicity, medical insurance, and the last systolic and diastolic BP measurements. Based on a patient’s primary address, we determined census tract-level neighborhood socioeconomic disadvantage indicators: the Area Deprivation Index (ADI, national-level percentile), based on the 2016‐2020 American Community Survey, and the Centers for Disease Control and Prevention’s Social Vulnerability Index (version 2020; overall score, national-level percentile) [18,19]. Health care–related predictors included the presence of individual Elixhauser Comorbidities, antihypertensive drug classes prescribed, and number of visits within different clinical settings [20]. We considered several biological measurements including total cholesterol, high-density lipoprotein cholesterol, and low-density lipoprotein cholesterol, triglycerides, and creatinine, as well as weight and BMI. BP measurements in the past 1 year of index visit were aggregated into prediction features (eg, count of BP readings ≥140/90 mm Hg). A detailed description of all prediction features can be found in Table S3 in Multimedia Appendix 1.

### Statistical Analyses

We calculated the required sample size of a validation dataset so that the 95% CI for validation C-statistics had a width of 0.05 or less [21]. A sample size of 11,520 patients is adequate for an outcome prevalence between 5%‐50% and a C-statistics between 0.6‐0.8.

We randomly split the dataset into a training set and an internal validation set according to an 80:20 ratio. We performed median imputation for numerical variables and used imputed median values from the training set for subsequent imputation with the validation set. To handle considerable missingness with laboratory tests, we categorized the variables using standard cutoff values for their normal ranges and applied the “missing” category to the variable when the value was not available. Once categorical variables were 1-hot encoded, we performed data standardization, subtracting the variables by the sample mean then dividing by the sample SD. No variable selection procedure was conducted. Further, 4 machine learning frameworks were considered: (L2) regularized logistic regression, multilayer perceptron (with 1 hidden layer), gradient boosting machines, and random forest. For hyperparameter tuning, we used grid search strategy with discrimination power (C-statistic) as the selection criteria and performed 10-fold cross-validation on the training set. Hyperparameter-tuned models were identified, one for each modeling framework, and further compared on their cross-validation performance for the final model.

For internal validation, in addition to discrimination, we assessed calibration performance of the 2 final models, one for each outcome, using smoothed calibration curves, estimated with generalized additive models. Based on the calibration curves, we computed the average (ie, integrated calibration index [ICI]), median (E50), 90th percentile (E90) of the absolute difference between expected event rate and predicted risk to summarize calibration errors [22]. We computed 95% CIs using standard methods for C-statistics (DeLong method) and calibration metrics (simulation-based inference) [23]. Additionally, we reported sensitivity, specificity, and positive or negative predictive values and 95% CIs (using exact binomial method) with respect to different decision thresholds. We performed decision curve analysis with the validation set to assess the models’ net benefit in comparison with the default policies, that is, treating all and treating no patients [24]. We evaluated net benefit within a range of probability decision threshold, which was 50%‐70% for the outcome sustained, uncontrolled hypertension and ≤20% for the outcome hypertensive crisis.

Finally, we carried out internal-external cross-validation (IECV) at the county level to examine the final models’ predictive ability in new patient cohorts, using the existing data [25]. With this approach, we used data from patients receiving care within a given county to validate models developed with the data of all other patients. For reliable validation, we only validated the counties yielding sufficient sample size of at least 200 events [26]. Using random-effect meta-analyses, we estimated the pooled performance measures, that is, the C-statistic, and ICI, across validations. We assessed heterogeneity across validations using the SD of the random effect, denoted as τ, and a chi-square test with a significance level of .05.

Model building was performed using Python (Python Software Foundation, eg, “scikit-learn” package). Other analyses were conducted using R (R Foundation, eg, “pROC,” “pmcalibration,” and “meta” packages).

### Ethical Considerations

The Atrium Health institutional review board approved our research protocol. Informed consent was waived as there were no more than minimal risks to study participants. The study data will not be made available publicly to ensure patient confidentiality and privacy.

## Results

### Patient Characteristics

This study’s cohort consisted of 142,897 patients, almost all of whom received care in 13 North Carolina counties and 1 South Carolina county in the year 2018. The patients were 72.33% (n=103,361) White and 22.52% (n=32,174) Black, had a median age of 61 (IQR 49-71) years and a median national-level ADI ranking of 55 (IQR 35-73). All patients were observed with known sustained, uncontrolled hypertension and hypertensive crisis status during follow-up period. The observed prevalence of sustained, uncontrolled hypertension and hypertensive crisis were 41.67% (n=59,547) and 4.53% (n=6,470), respectively. Across the counties, there were notable racial and socioeconomic differences (Table 1). Except for 1 county, the percentage of White patients ranged between 62.64% and 93.13% and the median of ADI ranking ranged between 39 and 88. The observed prevalence of outcomes among the counties ranged between 37.35% and 47.26% for sustained, uncontrolled hypertension, and, except for 1 county, between 3.89% and 6.06% for hypertensive crisis.

**Table 1.** Patient characteristics.

Location of care, county (sample size)	ADI^a^, median (IQR)	Age, median (IQR)	Female, n (%)	White, n (%)	Black, n (%)	SUHTN^b^, n (%)	HC^c^, n (%)
Overall(n=142,897)	55 (35-73)	61 (49-71)	85,113 (59.56)	103,361 (72.33)	32,174 (22.52)	59,547 (41.67)	6470 (4.53)
Mecklenburg(n=64,811)	44 (25-65)	60 (49-71)	39,347 (60.71)	40,595 (62.64)	19,746 (30.47)	26,338 (40.64)	2704 (4.17)
Cabarrus(n=31,403)	57 (42-72)	61 (49-71)	18,421 (58.66)	25,175 (80.17)	4891 (15.57)	13,854 (44.12)	1567 (4.99)
Union(n=9882)	54 (43-74)	60 (49-71)	5569 (56.35)	7400 (74.88)	1873 (18.95)	3963 (40.10)	443 (4.48)
York(n=8963)	51 (39-68)	61 (49-72)	5319 (59.34)	6991 (78.00)	1652 (18.43)	3694 (41.21)	384 (4.28)
Cleveland(n=6866)	78 (71-85)	63 (52-73)	4486 (65.33)	5347 (77.88)	1410 (20.54)	2918 (42.50)	416 (6.06)
Lincoln(n=4230)	71 (63-77)	63 (51-72)	2362 (55.84)	3852 (91.06)	266 (6.29)	1999 (47.26)	226 (5.34)
Gaston(n=3756)	67 (50-80)	60 (49-70)	1941 (51.68)	3145 (83.73)	507 (13.50)	1403 (37.35)	146 (3.89)
Stanly(n=2478)	68 (58-78)	64 (51-74)	1524 (61.50)	2148 (86.68)	304 (12.27)	1116 (45.04)	135 (5.45)
Iredell(n=2411)	39 (21-57)	70 (59-77)	1159 (48.07)	2169 (89.96)	148 (6.14)	953 (39.53)	96 (3.98)
Rutherford(n=1922)	82 (66-88)	67 (57-75)	1163 (60.51)	1645 (85.59)	245 (12.75)	771 (40.11)	82 (4.27)
Burke(n=1462)	79 (72-83)	58 (45-69)	1113 (76.13)	1332 (91.11)	91 (6.22)	608 (41.59)	52 (3.57)
Caldwell(n=1324)	84 (72-90)	64 (52-73)	728 (54.98)	1233 (93.13)	72 (5.44)	521 (39.35)	51 (3.85)
Rowan(n=1121)	69 (63-78)	59 (49-69)	635 (56.65)	868 (77.43)	219 (19.54)	500 (44.60)	45 (4.01)
Anson(n=625)	88 (80-96)	58 (50-69)	362 (57.92)	186 (29.76)	425 (68.00)	272 (43.52)	53 (8.48)

a ADI: Area Deprivation Index.

b SUHTN: sustained, uncontrolled hypertension.

c HC: hypertensive crisis.

### Final Models

For each prediction problem, the hyperparameter-tuned models from different modeling frameworks achieved practically equivalent 10-fold cross-validated C-statistics of around 0.71‐0.72 for the outcome sustained, controlled hypertension and 0.79‐0.80 for hypertensive crisis, respectively (Table 2). Given that the L2-regularized logistic regression (LR) was a simpler and more computationally efficient framework, we selected the hyperparameter-tuned LR models for training and final validations. Additionally, we examined the relative variable importance in a trained LR model via the magnitude of predictor coefficients and investigated each model’s top 10 variables (Table S1 in Multimedia Appendix 1). Notably, increases in systolic BP at index visit, the number of encounters with systolic BP≥140 mm Hg and the number of encounters with BP≥140/90 mm Hg in the past 1 year, age, as well as a prior diagnosis of hypertension and higher ADI ranking, predicted both higher risk of sustained, uncontrolled hypertension and hypertensive crisis.

**Table 2.** Optimized hyperparameters and 10-fold cross-validated C-statistics of the hyperparameter-tuned models.

Framework and hyperparameter options^a^		Sustained, uncontrolled hypertension		Hypertensive crisis	
		Optimal value	Cross-validated C-stat (SE)	Optimal value	Cross-validated C-stat (SE)
**L2 regularized logistic regression**			0.713 (0.002)		0.793 (0.002)
C^b^: 0.001, 0.01, …, 1000	0.001	0.001
**Gradient boosting**			0.716 (0.001)		0.799 (0.002)
	n_estimators: 50, 100, 150	100		100	
	learning_rate: 0.05, 0.1, 0.2	0.2		0.2	
	max_depth: 3, 5, 8	5		3	
**Multilayer perceptron**			0.713 (0.002)		0.794 (0.002)
	hidden_layer_size: 5, 10, 20	5		5	
	learning_rate_init: 0.001, 0.01, 0.1	0.01		0.01	
	alpha^c^: 0.00001, 0.0001, 0.001	0.001		0.001	
**Random forest**			0.708 (0.002)		0.785 (0.003)
	n_estimators: 50, 100, 150	50		50	
	max_depth: 3, 5, 7	7		7	
	max_features^d^: “sqrt,” “log2,” none	“sqrt”		None	

a Hyperpameters in scikit-learn Python package.

b C: inverse of regularization strength.

c alpha: strength of L2 regularization.

d max_features: number of features (function of n_features) to consider when looking for best split.

### Internal and Internal-External Validations

**Figure 1.**
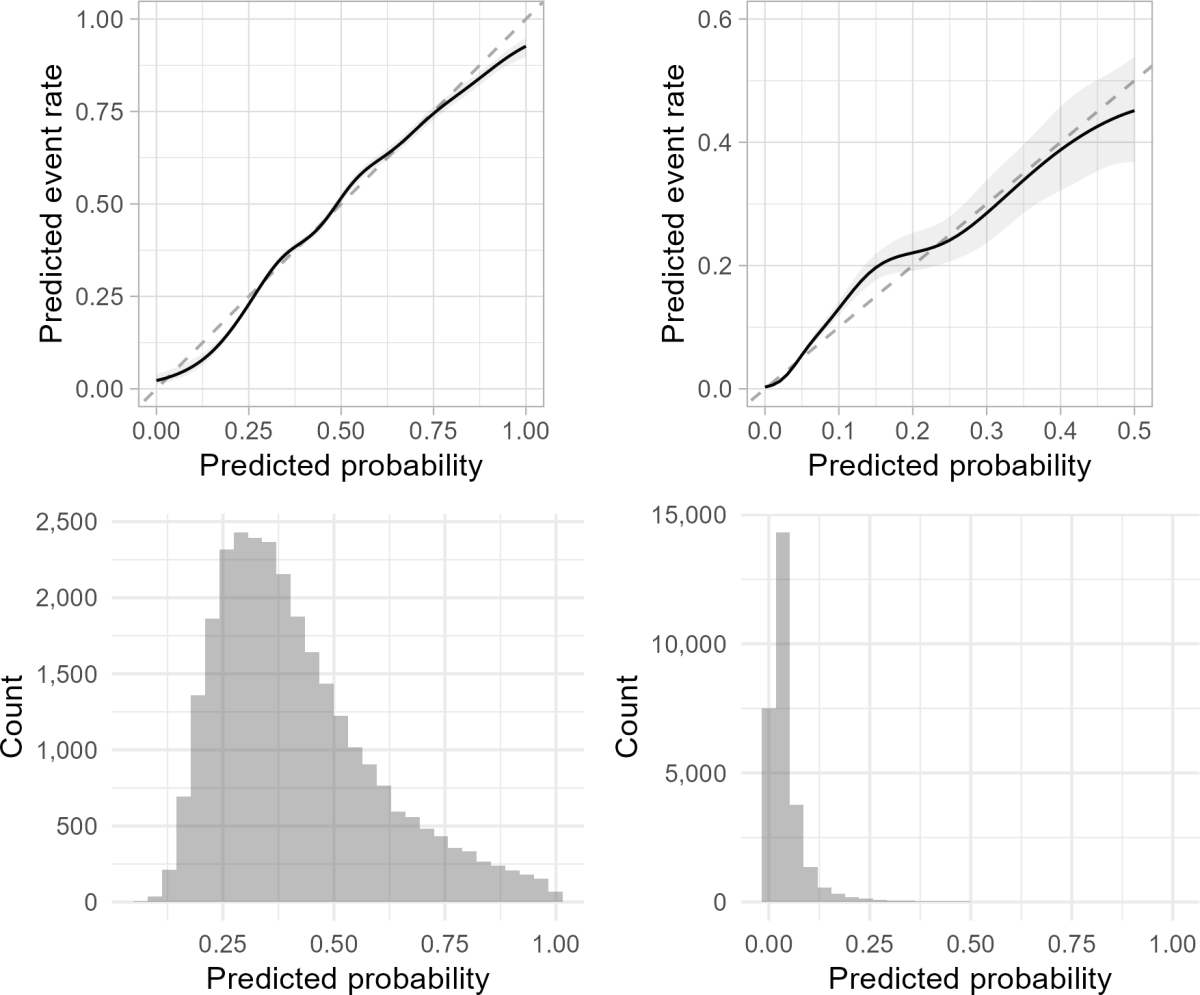
Smoothed calibration plots (with 95% CIs; top) and histograms showing distribution of the predicted probability (bottom) for the sustained, uncontrolled hypertension model (left) and the hypertensive crisis model (right) from internal validation.

**Figure 2.**
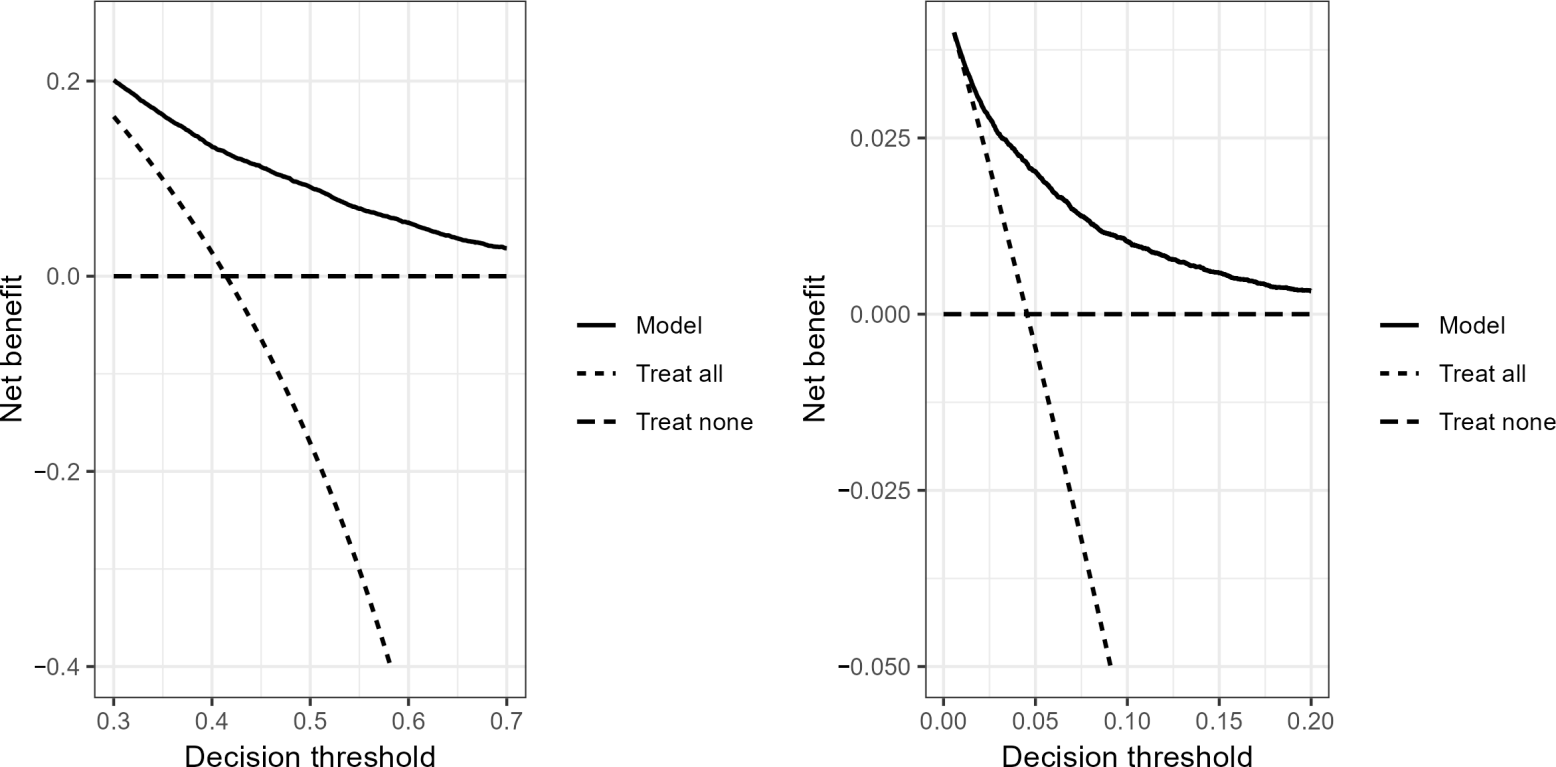
Decision curve analyses of the final models predicting sustained, uncontrolled hypertension (left) and hypertensive crisis (right) from internal validation.

In the internal validation dataset, the LR models achieved acceptable discrimination power for predicting sustained, uncontrolled hypertension with a C-statistic of 0.72 (95% CI 0.71‐0.72), and reasonably good discrimination power for predicting hypertensive crisis with a C-statistic of 0.81 (95% CI 0.79‐0.82). Both sustained, uncontrolled hypertension and hypertensive crisis models had accurate risk estimates with an ICI of 0.015 (95% CI 0.012‐0.020) and 0.009 (95% CI 0.007‐0.011), respectively. Other metrics (E50 and E90) and calibration curves can be examined in Table 3 and Figure 1. Sensitivity, specificity, and predictive values of the final models across potential decision thresholds were also reported in Table S2 in Multimedia Appendix 1. Further, in decision curve analyses, the models demonstrated higher net benefit than treat-all and treat-none policies within the ranges of plausible decision thresholds (Figure 2).

From IECV of the sustained, uncontrolled hypertension model, the pooled estimates were 0.70 (95% CI 0.69‐0.71) for C-statistic and 0.021 (95% CI 0.016‐0.026) for ICI; additionally, there was a small or moderate heterogeneity in C-statistic (τ=0.02, 95% CI 0.01‐0.03, *P*<.001) and a small heterogeneity in ICI (τ=0.01, 95% CI 0.01‐0.01, *P*<.001). From IECV of the hypertensive crisis model, the pooled estimates were 0.79 (95% CI 0.78‐0.81) for C-statistic and 0.007 (95% CI 0.005‐0.009) for ICI; across validations, variation in C-statistic was small or moderate (τ=0.01, 95% CI 0.00‐0.04, *P*=.004) and variation in ICI was small (τ=0.00, 95% CI 0.00‐0.01, *P*=.009).

**Table 3.** Discrimination and calibration performance (with 95% CIs) of the final models on training and internal validation datasets.

Model and dataset		Sample size (missing^a^) n	C-stat	ICI^b^	E50	E90
**Sustained, uncontrolled hypertension**						
	Training	114,317(7730)	0.71	0.015	0.015	0.030
	Internal validation	28,580(1959)	0.72(0.71-0.72)^c^	0.015(0.011‐0.020)^c^	0.009(0.005‐0.017)^c^	0.040(0.030‐0.047)^c^
**Hypertensive crisis**						
	Training	114,317(7730)	0.80	0.006	0.005	0.013
	Internal validation	28,580(1959)	0.81(0.79-0.82)^c^	0.009(0.007‐0.011)^c^	0.006(0.004‐0.007)^c^	0.021(0.014‐0.028)^c^

a Missing: number of observations containing any missing feature.

b ICI: integrated calibration index.

c 95% CI.

## Discussion

### Principal Results

Using a large, diverse (in terms of geography, race, and socioeconomic status) patient cohort in the Greater Charlotte area of the United States, we developed and validated risk models to predict sustained, uncontrolled hypertension and hypertensive crisis occurring within 1 year following an index ambulatory or outpatient encounter in which an uncontrolled BP reading was documented. Internal validation showed discrimination performance that was reasonably good for the hypertensive crisis model and lower but acceptable for the sustained, uncontrolled hypertension model. Overall, both models predicted the risks accurately (calibration performance) in the internal validation dataset and further demonstrated clinical utility through decision curve analyses. Using the existing data, IECV assessed the models’ predictive ability with new patient cohorts and showed that the models’ overall discrimination and calibration performance across validations were consistent with internal validation results. In addition, across validations, we observed small to moderate variation in discrimination performance and a small variation in calibration performance.

Our findings show that the risk of hypertensive crisis in patients with uncontrolled hypertension can be predicted well. The hypertensive crisis model, in particular, showed satisfactory performance to serve patients within Atrium Health’s North and South Carolina markets, and potentially patients from other nearby areas. Based on the model’s internal and internal-external validation performance, it also has the potential to be applicable to other health systems. For our final models to be further validated and used elsewhere, the complete specifications of the models can be found in Table S3 in Multimedia Appendix 1.

Therapeutic inertia (TI), that is, the failure of providers to initiate or intensify medication therapy when patients fail to achieve their treatment goals, is a well-known barrier to better clinical outcomes [27]. Among patients with hypertension, TI contributes to worse short- and long-term BP control [28,29]. While the causes of TI are multifactorial, interventions that include provider and patient education and leverage health care data to guide clinical decision-making at the point of care are promising approaches for reducing TI. Within this context, our prediction models can provide useful data to facilitate clinician-patient discussions on the potential need to intensify medications. Our expectation is that the use of these models will ultimately improve medication adherence and BP control in patients with uncontrolled hypertension. Future studies will be needed to assess the models’ clinical impacts to support implementation into routine clinical care.

While models such as ours are becoming increasingly more common as clinical decision support tools, several challenges to implementation can be expected. First, there is a need to continuously monitor for potential drifts from the models’ expected behaviors, at regular intervals [30]. This may include monitoring for changes in predictive performance, model usefulness, patient population, and predictor data being applied to the models. Substantial efforts may be required for monitoring, investigation of model issues, and model updating. Additionally, based on the 5 rights of clinical decision support framework (ie, right information, right person, right format, right channel, and right time) other challenges can be foreseen [31]. For example, alert fatigue is an issue where a high volume of alerts can overwhelm users and result in total disregard of the information provided. There is also a need to build clinical trust through proper presentation of the models’ facts to end users, such as the approved use case, potential risks and benefits of the model, and validation data on performance and clinical utility. All of these factors require careful attention in implementation studies to guide the proper use of the models in practice.

Our study also found that even with a comprehensive set of EHR-based predictors, predicting sustained, uncontrolled hypertension remains a challenging problem. The marginally acceptable discrimination performance results indicate a need to improve further our ability to predict this outcome. As we already attempted complex machine learning methods and a large sample size, more powerful predictors are needed to improve predictive performance. One future direction is to increase our ability to monitor and collect BP data, as BP-related predictors had relatively large impacts on risk scores. Wearable devices, such as fitness trackers, for example, are increasingly popular and can be used to collect BP data and support BP monitoring and management [32].

### Limitations

Our study has several limitations. First, our models, while adequate, have room for improvement by adding important predictor variables that were either unavailable or not considered. For example, modifiable risk factors, such as lifestyle behaviors, medication adherence, and medication dosing, are known to be associated with BP control, but these data were either not accessible from the EHR or came from unstructured data sources (eg, clinical notes), which can be challenging to process for prediction. Second, because patients without a BP reading in the prediction window were excluded, and models can only be developed and validated for patients who had some follow-up BP measurements, there is a risk for bias. Fortunately, a relatively small number of patients had no BP reading during the follow-up period, thus, the extent of bias, if any, can be deemed minor. Third, the models were developed for patients on 4 or less antihypertensive drug classes and may not generalize beyond this patient group. Fourth, additional prospective studies are needed to understand how our models would perform when implemented in real-world clinical settings.

### Comparison With Prior Work

Our study presented novel applications of predictive modeling to the area of hypertension management. From a design perspective, the prediction outcomes, targeted patient cohort, and intended use case were carefully chosen to optimize models’ usefulness for managing hypertension. We noted that while little research was performed about predicting clinically important hypertension states in patients with hypertensiveness, a much larger amount of literature was devoted toward predicting hypertension onset in the general population [11-13]. Comparing with other hypertension prediction studies, ours made use of a relatively diverse set of predictors, including EHR-based data that were not often considered, such as usage of different health services, drug classes prescribed, and social determinants of health [11]. In terms of performance, the C-statistics in our models were similar to those from existing prediction models of hypertension onset, which were between 0.63‐0.84 according to a meta-analysis [11]. A more recent, published prediction model of uncontrolled hypertension demonstrated a C-statistics of 0.76 [16]. Finally, a large and geographically diverse patient sample allowed us to assess (weak) generalizability through IECV, as well as to assess internal validity with confidence. In contrast, the vast majority of hypertension prediction studies were only internally validated and had smaller sample sizes [11].

### Conclusions

We developed and validated risk models for sustained, uncontrolled hypertension, and hypertensive crisis, within 1 year of an index visit showing an uncontrolled BP reading. The hypertensive crisis risk model showed good predictive performance with internal validation and new patient cohorts during IECV. This model could be prospectively validated as a next step. If validity and clinical utility are confirmed, it could then be used within our health system, and potentially elsewhere, as a public health surveillance tool for early detection of hypertensive crises and for supporting physicians with treatment decisions. Further efforts are required to improve the ability to predict sustained, uncontrolled hypertension, particularly by adding and improving predictor variables.

## Supplementary material

10.2196/58732 Multimedia Appendix 1 Additional tables.

## Abbreviations

ADI: Area Deprivation Index
BP: blood pressure
EHR: electronic health record
ICI: integrated calibration index
IECV: internal-external cross-validation
LR: logistic regression
TI: therapeutic inertia
