# Supplementary material for: Predictive Models for Sustained, Uncontrolled Hypertension and Hypertensive Crisis Based on Electronic Health Record Data: Algorithm Development and Validation
Source: JMIR Med Inform. 2024 Oct 28;12:e58732. doi: 10.2196/58732 (PMC11533385; doi:10.2196/58732)
Supplement: Multimedia Appendix 1 [file medinform-v12-e58732-s001.docx]

Multimedia Appendix 1

Table S1: Variable importance (top 10) in the final models (L2-regularized logistic regression), measured by a variable’s coefficient relative to the largest coefficient magnitude.

| Rank | Sustained, uncontrolled hypertension model | | Hypertensive crisis model | |
| --- | --- | --- | --- | --- |
|  |  | |  | |
|  | Predictor | (Relative) coefficient | Predictor | (Relative) coefficient |
| 1 | 1-year-look-back encounters with BP ≥ 140/90 mm Hg | 1.00 (ref) | Systolic BP at index | 1.00 (ref) |
| 2 | Systolic BP at index | 0.96 | Age | 0.40 |
| 3 | 1-year-look-back encounters with SBP ≥ 140 mm Hg | 0.67 | 1-year-look-back encounters with SBP ≥ 140 mm Hg | 0.34 |
| 4 | Age | 0.43 | Comorbidity (Hypertension without Complications) | 0.31 |
| 5 | Comorbidity (Hypertension without Complications) | 0.36 | 1-year-look-back ambulatory encounters | -0.27 |
| 6 | Diastolic BP at index | 0.33 | 1-year-look-back encounters with BP ≥ 140/90 mm Hg | 0.22 |
| 7 | Body mass index | 0.30 | Insurance (Medicare) | 0.18 |
| 8 | Race (Black/African American) | 0.24 | 1-year-look-back outpatient encounters | -0.17 |
| 9 | Comorbidity (Solid Tumor without Metastasis) | 0.18 | Area Deprivation Index Rank | 0.15 |
| 10 | Area Deprivation Index Rank | 0.17 | 1-year-look-back encounters with SBP ≥ 180 mm Hg | 0.15 |

Table S2: Sensitivity, specificity, and predictive values from internal validation of the final models

| Model | Probability threshold | Sensitivity  (95% CI) | Specificity  (95% CI) | Positive predictive value (95% CI) | Negative predictive value (95% CI) |
| --- | --- | --- | --- | --- | --- |
|  |  |  |  |  |  |
| Sustained, uncontrolled hypertension | 30% | 0.85 (0.84-0.85) | 0.40 (0.39-0.41) | 0.50 (0.49-0.51) | 0.79 (0.78-0.80) |
|  | 35% | 0.74 (0.74-0.75) | 0.55 (0.54-0.56) | 0.54 (0.53-0.55) | 0.75 (0.74-0.76) |
|  | 40% | 0.63 (0.62-0.64) | 0.67 (0.67-0.68) | 0.58 (0.57-0.58) | 0.72 (0.71-0.73) |
|  | 42% (prevalence) | 0.59 (0.58-0.60) | 0.72 (0.71-0.72) | 0.59 (0.59-0.60) | 0.71 (0.70-0.72) |
|  | 45% | 0.53 (0.50- 0.54) | 0.77 (0.77-0.78) | 0.62 (0.61-0.63) | 0.70 (0.69-0.71) |
|  | 50% | 0.44 (0.43-0.45) | 0.85 (0.84-0.85) | 0.67 (0.66-0.68) | 0.68 (0.67-0.69) |
|  | 55% | 0.35 (0.34-0.36) | 0.89 (0.89-0.90) | 0.70 (0.69-0.71) | 0.66 (0.65-0.67) |
|  | 60% | 0.28 (0.28-0.29) | 0.93 (0.92-0.93) | 0.74 (0.73-0.75) | 0.65 (0.64-0.65) |
|  | 65% | 0.22 (0.22-0.23) | 0.95 (0.95-0.95) | 0.76 (0.75-0.78) | 0.63 (0.63-0.64) |
|  | 70% | 0.18 (0.17-0.18) | 0.97 (0.97-0.97) | 0.79 (0.78-0.81) | 0.62 (0.62-0.63) |
| Hypertensive crisis | 2.5% | 0.91 (0.89-0.92) | 0.45 (0.45-0.46 | 0.07 (0.07-0.08) | 0.99 (0.99-0.99) |
|  | 5% (prevalence) | 0.70 (0.67-0.72) | 0.77 (0.77-0.78) | 0.13 (0.12-0.14) | 0.98 (0.98-0.98) |
|  | 7.5% | 0.50 (0.47-0.53) | 0.89 (0.88-0.89) | 0.17 (0.16-0.19) | 0.97 (0.97-0.98) |
|  | 10% | 0.38 (0.36-0.41) | 0.93 (0.93-0.94) | 0.21 (0.20-0.23) | 0.97 (0.97-0.97) |
|  | 12.5% | 0.30 (0.27-0.32) | 0.96 (0.96-0.96) | 0.25 (0.23-0.27) | 0.97 (0.96-0.97) |
|  | 15% | 0.24 (0.22-0.27) | 0.97 (0.97-0.97) | 0.27 (0.25-0.30) | 0.96 (0.96-0.97) |
|  | 17.5% | 0.20 (0.18-0.22) | 0.98 (0.97-0.98) | 0.29 (0.26-0.32) | 0.96 (0.96-0.96) |
|  | 20% | 0.16 (0.14-0.19) | 0.98 (0.98-0.98) | 0.31 (0.27-0.34) | 0.96 (0.96-0.96) |

Table S3: Coefficients of the predictors in the final models (L2-regularized logistic regression).

| No. | Predictor | Coefficient (SUHTN^a^ model) | Coefficient (HC model^b^) |
| --- | --- | --- | --- |
|  |  |  |  |
| 0 | 1 (Intercept) | -6.6033 | -10.6352 |
| 1 | Age at index visit | 0.0086 | 0.0124 |
| 2 | Sex (Male indicator) | -0.0174 | -0.1393 |
| 3 | Race (White/Caucasian indicator) | 0.0159 | -0.0222 |
| 4 | Race (Black/African American indicator) | 0.1876 | 0.1490 |
| 5 | Area Deprivation Index ACS5 2016-2020 (National-level ranking, range: 0-100) | 0.0024 | 0.0033 |
| 6 | Social Vulnerability Index version 2020 (National-level ranking of the overall score, range: 0-1) | 0.0001 | -0.0005 |
| 7 | Body mass index (BMI) | 0.0128 | 0.0022 |
| 8 | Weight (lbs) | 0.0000 | -0.0010 |
| 9 | Medical insurance (Medicaid indicator) | -0.0130 | 0.0337 |
| 10 | Medical insurance (Medicare indicator) | 0.0899 | 0.1772 |
| 11 | Medical insurance (Commercial indicator) | -0.0024 | -0.1043 |
| 12 | Medical insurance (Self-pay indicator) | 0.0302 | 0.1726 |
| 13 | Systolic BP at index visit (the last measurement) (mm Hg) | 0.0248 | 0.0400 |
| 14 | Diastolic BP at index visit (the last measurement) (mm Hg) | 0.0102 | 0.0033 |
| 15 | 1-year-look-back number of encounters with systolic BP ≥ 140 mm Hg | 0.1317 | 0.1025 |
| 16 | 1-year-look-back number of encounters with diastolic BP ≥ 90 mm Hg | 0.0465 | 0.0084 |
| 17 | 1-year-look-back number of encounters with BP ≥ 140/90 mm Hg | 0.1838 | 0.0610 |
| 18 | 1-year-look-back number of encounters with systolic BP ≥ 180 mm Hg | 0.0604 | 0.3183 |
| 19 | 1-year-look-back number of encounters with diastolic BP ≥ 120 mm Hg | -0.0868 | 0.3220 |
| 20 | 1-year-look-back number of encounters with BP ≥ 180/120 mm Hg | 0.0723 | 0.2762 |
| 21 | 1-year-look-back number of ambulatory encounters | 0.0031 | -0.0083 |
| 22 | 1-year-look-back number of outpatient encounters | 0.0049 | -0.0186 |
| 23 | 1-year-look-back number of emergency department encounters | 0.0221 | 0.0111 |
| 24 | 1-year-look-back number of inpatient encounters | 0.0095 | 0.0652 |
| 25 | 1-year-look-back number of observation encounters | 0.0002 | -0.0031 |
| 26 | (1-year-look-back) Comorbidity (AIDS/HIV indicator) | 0.4406 | 0.4153 |
| 27 | Comorbidity (Alcohol Abuse indicator) | 0.1227 | 0.1825 |
| 28 | Comorbidity (Blood Loss Anemia indicator) | 0.1088 | -0.0335 |
| 29 | Comorbidity (Anemia Deficiency indicator) | 0.1348 | 0.0937 |
| 30 | Comorbidity (Cardiac Arrhythmia indicator) | -0.0064 | -0.0434 |
| 31 | Comorbidity (Chronic Pulmonary Disease indicator) | 0.1259 | -0.0001 |
| 32 | Comorbidity (Coagulopathy indicator) | 0.0356 | -0.1226 |
| 33 | Comorbidity (Congestive Heart Failure indicator) | -0.0507 | 0.0779 |
| 34 | Comorbidity (Depression indicator) | 0.0333 | -0.0389 |
| 35 | Comorbidity (Diabetes with complications indicator) | 0.1082 | 0.1081 |
| 36 | Comorbidity (Diabetes without complications indicator) | 0.0136 | 0.0776 |
| 37 | Comorbidity (Drug Abuse indicator) | -0.0483 | 0.2008 |
| 38 | Comorbidity (Fluid and Electrolyte Disorders indicator) | 0.0910 | 0.2066 |
| 39 | Comorbidity (Hypertension with complications indicator) | -0.0009 | 0.0744 |
| 40 | Comorbidity (Hypertension without complications indicator) | 0.2350 | 0.3061 |
| 41 | Comorbidity (Hypothyroidism indicator) | -0.0138 | -0.0187 |
| 42 | Comorbidity (Liver Disease indicator) | 0.1208 | -0.0641 |
| 43 | Comorbidity (Lymphoma indicator) | 0.4149 | 0.1007 |
| 44 | Comorbidity (Metastatic Cancer indicator) | 0.3447 | -0.0278 |
| 45 | Comorbidity (Solid Tumor without Metastatic indicator) | 0.1990 | 0.0202 |
| 46 | Comorbidity (Obesity indicator) | -0.0118 | -0.0540 |
| 47 | Comorbidity (Other Neurological Disorders indicator) | -0.0004 | -0.0642 |
| 48 | Comorbidity (Paralysis indicator) | -0.0980 | 0.0275 |
| 49 | Comorbidity (Peptic Ulcer Disease (Excluding Bleeding) indicator) | 0.0294 | -0.1677 |
| 50 | Comorbidity (Peripheral Vascular Disease indicator) | 0.0301 | 0.2709 |
| 51 | Comorbidity (Psychoses indicator) | -0.1456 | -0.2439 |
| 52 | Comorbidity (Pulmonary Circulation Disorder indicator) | 0.0714 | 0.0629 |
| 53 | Comorbidity (Renal Failure indicator) | 0.0768 | 0.1383 |
| 54 | Comorbidity (Rheumatoid Arthritis Collagen Vascular Disease indicator) | 0.1500 | 0.0156 |
| 55 | Comorbidity (Valvular Disease indicator) | 0.1203 | 0.0708 |
| 56 | Comorbidity (Weight Loss indicator) | 0.0197 | 0.0563 |
| 57 | Maximum of cholesterol values in the last 6 months of index visit ≤ 200 mg/dL (indicator; also, 0 if data not available) | -0.0346 | -0.0131 |
| 58 | Maximum of cholesterol values in the last 6 months of index visit > 200 mg/dL (indicator; also, 0 if data not available) | -0.0166 | -0.0719 |
| 59 | Minimum of HDL values in the last 6 months of index visit ≤ 60 mg/dL (indicator; also, 0 if data not available) | 0.0100 | -0.0273 |
| 60 | Minimum of HDL values in the last 6 months of index visit > 60 mg/dL (indicator; also, 0 if data not available) | -0.0464 | -0.0774 |
| 61 | Maximum of LDL values in the last 6 months of index visit ≤ 100 mg/dL (indicator; also, 0 if data not available) | -0.0011 | -0.1127 |
| 62 | Maximum of LDL values in the last 6 months of index visit > 100 mg/dL (indicator; also, 0 if data not available) | 0.0429 | 0.0704 |
| 63 | Maximum of triglycerides values in the last 6 months of index visit ≤ 150 mg/dL (indicator; also, 0 if data not available) | -0.0053 | -0.0359 |
| 64 | Maximum of triglycerides values in the last 6 months of index visit > 150 mg/dL (indicator; also, 0 if data not available) | 0.0483 | -0.0095 |
| 65 | Maximum of creatinine values in the last 12 months of index visit ≤ 0.95 mg/dL (indicator; also, 0 if data not available) | 0.0242 | 0.0290 |
| 66 | Maximum of creatinine values in the last 12 months of index visit > 0.95 mg/dL (indicator; also, 0 if data not available) | 0.0638 | 0.1892 |
| 67 | (1-year-look-back, including index visit) Antihypertensive Medication Class (Angiotensin II antagonists indicator) | 0.0706 | 0.1457 |
| 68 | Antihypertensive Medication Class (angiotensin-converting enzyme inhibitors (ACEIs) indicator) | -0.0308 | 0.1120 |
| 69 | Antihypertensive Medication Class (Beta blockers indicator) | 0.0244 | 0.1183 |
| 70 | Antihypertensive Medication Class (Alpha-1 blockers indicator) | 0.0770 | 0.2047 |
| 71 | Antihypertensive Medication Class (Beta blockers with intrinsic sympathomimetic activity indicator) | 0.2169 | 0.5583 |
| 72 | Antihypertensive Medication Class (Combination indicator) | -0.0960 | 0.0433 |
| 73 | Antihypertensive Medication Class (Thiazide diuretics indicator) | -0.0068 | -0.0083 |
| 74 | Antihypertensive Medication Class (Potassium-sparing diuretics indicator) | 0.5449 | -0.8046 |
| 75 | Antihypertensive Medication Class (Aldosterone receptor blockers indicator) | -0.0076 | -0.0294 |
| 76 | Antihypertensive Medication Class (Combined alpha- and beta blockers indicator) | -0.0236 | 0.0014 |
| 77 | Antihypertensive Medication Class (Central alpha-2 agonists and other centrally acting drugs indicator) | 0.1301 | 0.5534 |
| 78 | Antihypertensive Medication Class (Calcium channel blockers (CCBs)-nondihydropyridines indicator) | 0.0572 | 0.0446 |
| 79 | Antihypertensive Medication Class (Calcium channel blockers (CCBs)-dihydropyridines indicator) | 0.0420 | -0.1044 |
| 80 | Antihypertensive Medication Class (Loop diuretics indicator) | -0.0458 | 0.0369 |
| 81 | Antihypertensive Medication Class (Direct vasodilators indicator) | 0.1071 | 0.2020 |

^a^SUHTN: sustained, uncontrolled hypertension.

^b^HC: hypertensive crisis.
